# Management of Pediatric Humeral Supracondylar Fractures in a Referral Center From a Developing Country: A Comparison With American Academy of Orthopaedic Surgeons (AAOS) Guidelines

**DOI:** 10.7759/cureus.44430

**Published:** 2023-08-31

**Authors:** Alberto Daniel Navarro Vergara, Alberto Navarro Fretes, Rafael Aníbal Arréllaga Alonso, Maria Mercedes Medina Villate

**Affiliations:** 1 Orthopedics and Traumatology, Hospital de Trauma “Manuel Giagni”, Asunción, PRY; 2 Orthopedics and Traumatology, Hospital de Especialidades Quirúrgicas Ingavi del Instituto de Previsión Social (IPS), Asunción, PRY; 3 Orthopedics and Traumatology, Universidad del Norte, Asunción, PRY; 4 Pediatric Orthopedics, Hospital de Especialidades Quirúrgicas Ingavi del Instituto de Previsión Social (IPS), Asunción, PRY; 5 Pediatrics Service, Hospital de Trauma “Manuel Giagni”, Asunción, PRY

**Keywords:** complications, humerus, treatment, supracondylar humeral fracture, pediatrics, fracture

## Abstract

Introduction

Supracondylar fractures are the predominant type of pediatric elbow fractures. The usual mechanism of injury is falling over the hand with the elbow extended and the hand open. The management of these fractures encompasses a range of treatment options, and their goal is to recover the anatomy and achieve a stable contact area between them. There are some controversies on the management of these injuries mainly focused on those that present some degree of displacement. A review and analysis of the current treatment at our institution and a comparison with the guidelines suggested by the American Academy of Orthopaedic Surgeons (AAOS) for the treatment of these fractures in the pediatric population were performed.

Materials and methods

This was an observational, analytical, retrospective study of consecutive pediatric patients with displaced supracondylar humeral fracture treated at Hospital de Trauma “Manuel Giagni” in Asunción, Paraguay, from January 2016 to December 2021. Demographic and clinical data were assessed, and patients were clinically and radiologically followed for at least 12 months. The management of supracondylar humeral fractures at our hospital was compared with the guidelines suggested by the American Academy of Orthopaedic Surgeons (AAOS) by analyzing whether these guidelines were applied in each case. The mechanism of injury was divided into three groups, initial X-rays were measured, extension-type fractures were categorized into three groups, and the type of treatment was divided into two groups: non-operative and operative. Furthermore, trauma-related preoperative complications and postoperative complications were reported. Outpatient follow-up was performed for at least 12 months in all cases.

Results

Of the 843 patients analyzed, 71.5% were male, with a mean age of 5.6 years. It was observed that 57.5% of injuries were caused by falls on the same level. The most frequent type of injury was Gartland type III, accounting for 55% of the cases, and associated injuries were found in 4% of the cases. With regard to the type of treatment, 91.8% of patients were treated with closed reduction and percutaneous pin fixation. Complications on admission were found in 12% of the cases and late complications in 12% of the cases. Most patients (82%) had excellent Mayo Elbow Performance Score.

Conclusion

Supracondylar fractures were more frequent in males and in schoolchildren. Garland type III fractures were the most common type of injury. The treatment of choice was predominantly closed reduction and percutaneous pin fixation. The Mayo Elbow Performance Score was excellent in most patients. Our service, a referral center of a public hospital in a developing country, complies with the guidelines recommended by the AAOS.

## Introduction

Supracondylar humerus fractures account for almost 70% of elbow fractures in children [1]. According to numerous authors, its peak incidence occurs at 6-7 years of age [2,3], with currently no predominance of sex [4,5].

The typical mechanism of injury, which occurs in approximately 98% of cases, is a fall on an outstretched hand [2-6]. Supracondylar fractures usually present highly displaced fragments with no bone cortical contact, which sometimes may be accompanied by neurovascular injuries. Two factors should be considered to achieve proper management of this fracture: the location of the fracture line and the amount of displacement. Both factors can be used to classify the fracture to determine prognosis and actions to be taken [6,7]. The treatment options for these fractures are varied: immobilization without reduction, closed reduction and plaster cast, closed reduction followed by osteosynthesis through percutaneous pinning, and open reduction in some cases [7]. However, the common denominator is always achieving anatomical reduction of fragments and maintaining a stable contact area between them. In displaced fractures, close reduction and osteosynthesis with percutaneous pinning exhibit better results than non-surgical management [8].

The modified Gartland classification [4] is based on the degree of fragment displacement: type I (non-displaced fractures), type II (fractures with distal fragment displacement and intact posterior cortical hinge), and type III (fractures with complete displacement and without an intact cortical hinge). Type III is the most frequent presentation, with a twofold higher frequency than that of type II [2-4,8]. The discussion on the management of these fractures is mainly centered on injuries that present some degree of displacement (type II and III) [4] since there is full consensus on non-operative treatment [9]. Considering our experience, we proposed to review and analyze the standard of management of supracondylar humeral fractures compared to the guidelines suggested by the American Academy of Orthopaedic Surgeons (AAOS) for the treatment of this type of injuries in the pediatric population [9]. Because our experience was conducted in public service in Paraguay, a developing country, it may be hypothesized that there may be considerable differences in the comprehensive management of the disease under study with regard to international guidelines. Our results aim to create management guidelines for our country and for those with the same socioeconomic situation located in our region or in more remote locations.

## Materials and methods

This is an observational, analytical, retrospective study with consecutive pediatric patients with displaced supracondylar humeral fracture at Hospital de Trauma “Manuel Giagni” in Asunción, Paraguay (a pediatric trauma referral center), from January 2016 to December 2021. Patients with fractures caused by previous bone fragility or history of re-fractures were excluded from the study, as well as those with flexion-type fractures.

The demographic data of the study group were analyzed (sex, age, and seasonality), in addition to clinical data (mechanism of injury, affected side, type of injury, presence of open fractures, associated injuries, type of treatment, configuration of fixation, use of medial fixation, pre- and postoperative complications, and time elapsed from the trauma event to arrival at our service). Furthermore, all patients were clinically and radiographically followed for at least 12 months.

The management of supracondylar humeral fractures at our hospital was compared with the guidelines recommended by the AAOS [9] by analyzing whether these guidelines were applied in each case treated in our hospital.

The mechanism of injury was divided into three groups: fall on the same level (<30 cm), fall from height (>30 cm), and other mechanisms (traffic accidents/direct impacts). Injuries were classified as extension-type or flexion-type fractures according to the radiographs taken on admission to the service. Subsequently, injuries radiologically classified as extension-type fractures were categorized into three groups, based on the Wilkins modification of the Gartland classification [4]. Open fractures were classified according to the criteria described by Gustilo et al. [10]. The presence of associated injuries was reported considering the affected region of either the ipsilateral or the contralateral limb. Time elapsed (in days) from the trauma event to admission to our service was also assessed, assigning zero to cases that arrived at our service up to 24 hours after the event and day 1 onward to those that present more than 24 hours after the event.

The type of treatment was divided into two groups: non-operative and operative, with the first including cases that underwent placement of splint or plaster cast on the affected extremity without undergoing previous manipulation. Surgical treatments were categorized into closed and open procedures. The configuration of fixations was divided into two groups: medial fixation or only lateral. Preoperative findings related to trauma were recorded (distal sensory loss and hand pallor), as well as immediate postoperative complications, which included cases of reintervention, fixation failure, and vascular and nerve injuries. Outpatient follow-up was conducted at seven days, one month, three months, six months, and 12 months after the trauma event. Joint range of motion was recorded from three months after trauma onward, and residual functional limitations were assessed six and 12 months after trauma using the Mayo Elbow Performance Score, considering factors such as pain, range of motion, and stability (Table 1) [11].

**Table 1:** Mayo Elbow Performance Score Reproduced, with permission, from Schneeberger AG, Kösters MC, Steens W: Comparison of the subjective elbow value and the Mayo Elbow Performance Score. J Shoulder Elbow Surg. 2014, 23:308-12. 10.1016/j.jse.2013.11.018 [11].

Characteristic	Maximum score	Definition		Value
Pain	45	None		45
		Mild		30
		Moderate		15
		Severe		0
Range of motion	20	Range > 100		20
		Range from 50 to 100		15
		Range < 50		5
Stability	10	Stable		10
		Mildly stable		5
		Unstable		0
Daily function	25	Combing hair		5
		Eating		5
		Putting on shirt		5
		Putting on shoes		5
		Performing personal hygiene		5
Total	100		Excellent	>90
			Good	75-90
			Fair	60-75
			Poor	<75

Data required for investigation were collected using a form that was consecutively completed as cases were identified. Univariate statistical data analysis was conducted in Microsoft Excel (Microsoft Corporation, Redmond, WA, USA). Quantitative variables were expressed in absolute values and measures of central tendency, and qualitative variables in frequency and percentage tables, considering a statistical significance level of p < 0.05.

The present study complied with ethical guidelines and principles and was approved by the Research Ethics Committee and the General Direction of the Hospital de Trauma “Manuel Giagni” (number: 23/04-19ME1). Due to the retrospective nature of the study, patient informed consent was waived. The investigation was classified as harmless to humans. Patients’ personal data were not disclosed, and their medical records were not shared with individuals not belonging to the study group.

## Results

During the period of the study, 856 cases of supracondylar humeral fractures were treated at our institution. Of these, 13 flexion-type fractures were excluded. There were no cases of fractures due to previous bone fragility.

Of the 843 patients (Table 2), 604 (71.5%) were male, with a mean age of 5.6 years (ranging from one to 15 years old). The injury most often occurred in the summer (39%), followed by fall (24%), spring (22%), and winter (15%).

**Table 2:** Patients’ gender and season of trauma

Gender	Quantity	Percentage
Male	603	71.5%
Female	240	28.5%
Season	Quantity	Percentage
Summer	329	39%
Fall	202	24%
Spring	185	22%
Winter	127	15%

It was observed (Table 3) that 40% of the trauma events were caused by falls from height, 57.5% by falls at ground level (sports/domestic accidents), and 2.5% by other mechanisms, including traffic accidents or trauma. Moreover, 62% of the fractures occurred on the left side.

**Table 3:** Result type of mechanism of trauma and side of trauma

Trauma case	Percentage
Fall from height	40%
Fall on the same level	57.50%
Other mechanism	2.5%
Side of fracture	Percentage
Left	62%
Right	38%
Bilateral	0%

According to the modified Gartland classification [4], 55% of the cases were classified as type III, 46% as type II, and 4% as type I. Twenty-one (2.49%) patients had at least one open fracture. Sixteen open fractures were classified as Gustilo-Anderson type II and five as Gustilo-Anderson type IIIa. All open fractures were categorized as Gartland type III.

Associated injuries were found in 34 (4%) patients. Of these, there were 22 cases of floating elbow caused by injuries to the ipsilateral distal extremity of the forearm and 12 injuries to the contralateral upper extremity or other body regions.

With regard to the time elapsed from the trauma event to arrival at our service, 75% of patients presented to our service up to 24 hours after the event, whereas 12.5% presented on day 1 after the event, and 12.5% were referred to our service two days after the event or later.

**Figure 1:**
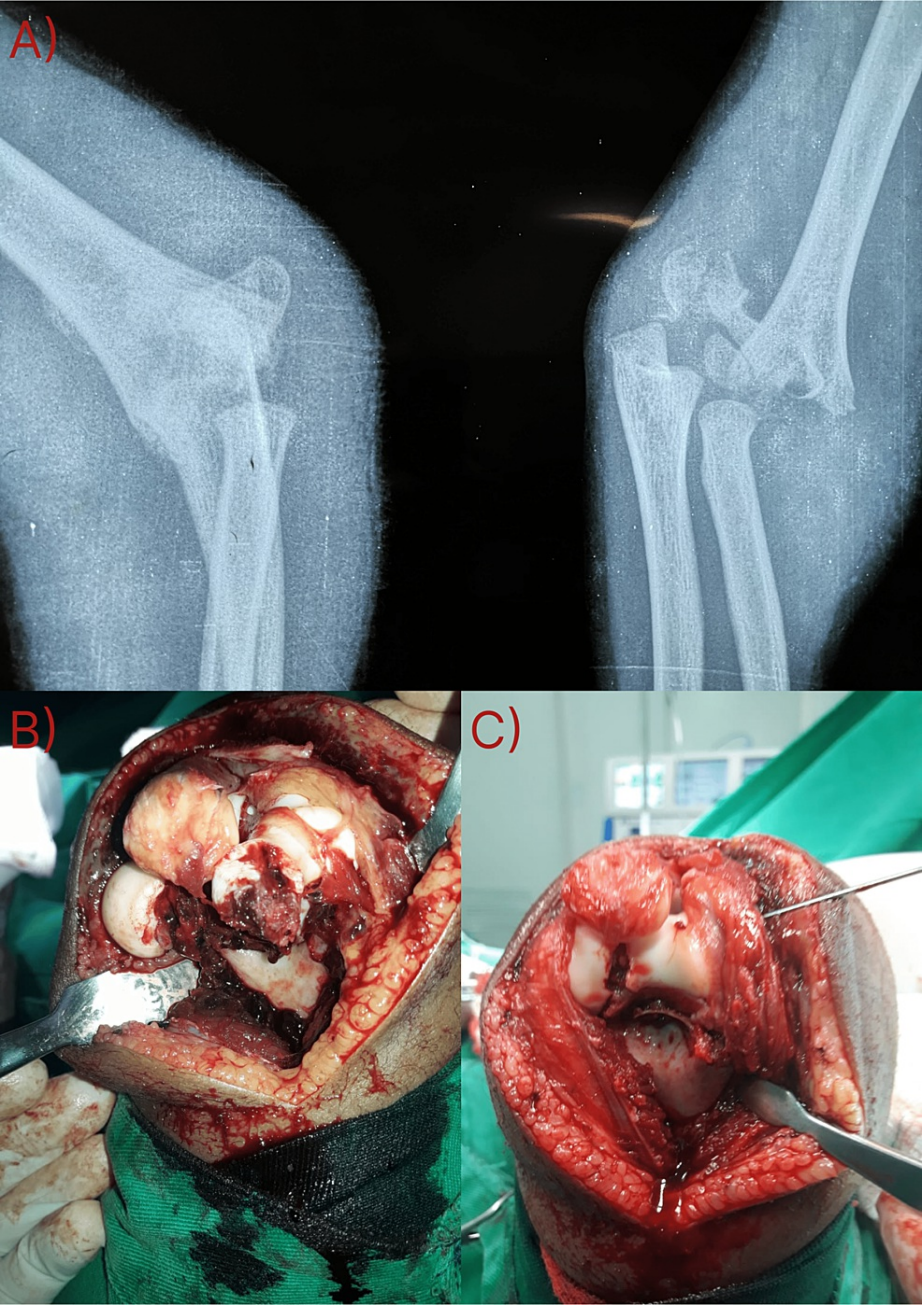
(A) Initial radiograph of the elbow showing a supracondylar humeral fracture with joint involvement, and (B and C) intraoperative image of a complex injury treated with anatomical reduction with lateral pinning

The vast majority of patients (91.8%) were treated with closed reduction and percutaneous pin fixation, whereas 4% were treated with immobilization with no previous manipulation, and 3.2% required open reduction (Figure 1).

Of the 800 cases requiring fixation, 548 (68.5%) underwent fixation with lateral-only pinning, and 252 (31.5%) were treated with medial column fixation with cross pins.

**Figure 2:**
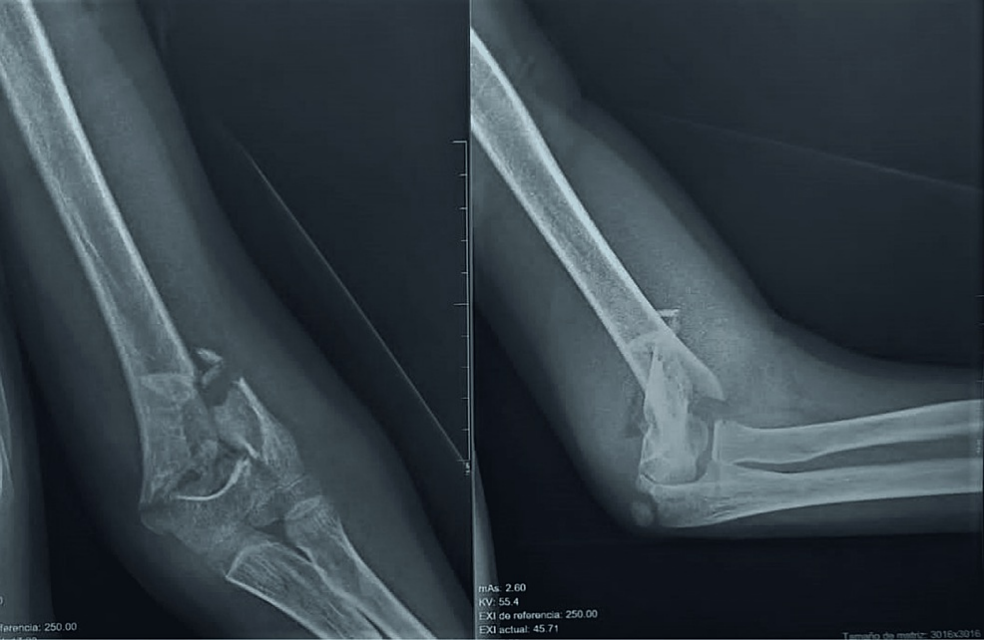
Radiographs showing left supracondylar humeral fracture, taken from an 11-year-old male patient with white hand sign and suspected compartment syndrome

There were 101 (12%) complications; 59 patients presented nerve complications and 42 had suspected vascular injuries due to distal pallor and absence of distal radial pulse on admission. Of the latter, two required vascular exploration after not showing improvement with reduction and fixation (Figure 2).

A neurological assessment revealed that 29 patients presented anterior interosseous nerve involvement, 20 presented radial nerve involvement, and 10 presented median nerve involvement. No cubital nerve injuries were reported on admission. None of the nerve injuries required surgical explorations during follow-up.

**Figure 3:**
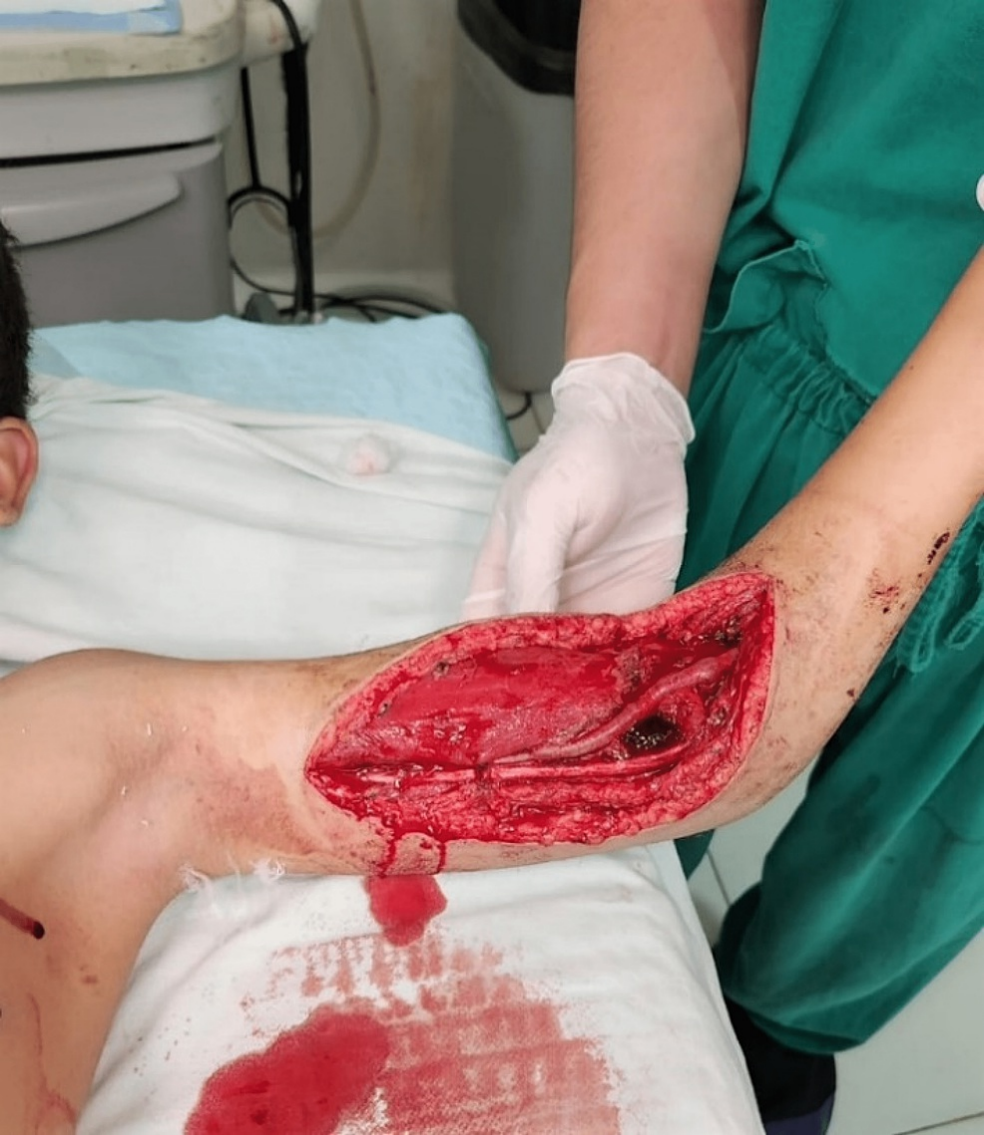
Photograph of the same patient as in Figure 2 showing open vascular bypass due to lack of soft tissue coverage

On admission, compartment syndrome was suspected in one patient who underwent fasciotomy and subsequently flap plastic surgery (Figure 3).

With regard to immediate postoperative complications, re-manipulation was required in 67 (8%) cases, and cubital neuropraxia was observed in 13 (1.54%) cases, all of which were related to a medial entry pin. These cases were evaluated at the follow-up outpatient visit and had a satisfactory outcome, with no need for intervention. No vascular complications or cases of compartment syndrome were reported postoperatively.

Late complications, such as angular deformity, were found in 101 (12%) patients, of whom 74% presented varus deformity of the elbow. Elbow rigidity at three months was observed in four (0.5%) cases. All patients were assessed using the Mayo Elbow Performance Score at six and 12 months after trauma, which was excellent in 82% of the cases, good in 12%, and fair or poor in 6%.

## Discussion

The present study showed that our findings on pediatric patients with humeral supracondylar fracture are similar to those described in the literature [2,3]. The predominance of this entity in males (71.5%) was also observed by Joshi et al. [12], who reported that, out of 700 patients, 56.7% were male. However, a study recently published by Poulios et al. [13] showed a slight predominance of females.

In a study of 159 pediatric patients with supracondylar humeral fracture, Barr [14] found that 53% were male, with a median age of six years and one month (ranging from one year to 14 years and four months), similar to the mean age described in the present study (5.6 years) and that in the study by Baidoo et al. [15]. Furthermore, a prospective investigation with 93 patients conducted by Cekanauskas et al. [16] found a mean age of 5.2 years.

Our findings in relation to seasonality were consistent with those by Barr [14], which showed that the incidence of supracondylar humeral fractures was significantly higher in the summer. Likewise, Pilla et al. [17] and Holt et al. [18] indicated that almost 60% of the fractures occur during school breaks, either during summer vacations or during spring break.

With regard to the mechanism of injury, there is a notorious predominance of extension-type injuries. In a study of 159 patients, Barr [14] found that 155 presented extension-type injuries, and Baidoo et al. [15] described that 96% of fractures were of the extension type. These findings are consistent with those of our study, which showed that flexion-type fractures are a rare entity, accounting for less than 2% of the cases. In relation to the affected side, the referenced studies coincide in showing a predominance of the non-dominant side [13-15]. The present study found that 62% of injuries occurred on the left side, in line with the results described by Poulios et al. [13] and Baidoo et al. [15] in their publications.

Cekanauskas et al. [16], in turn, reviewed and analyzed the treatment pattern of 93 patients, of which 63 were classified as Gartland type III, 23 as type II, four as type I, and six fractures were comminuted [16]. However, their study excluded flexion-type injuries. Poulios et al. [13] observed that 36 (32.14%) patients had type II fractures, 65 (58.03%) had type III fractures, and nine (8%) had type IV fractures, according to the new modified Gartland classification [4-7].

In the study by Barr [14], 65 (41%) patients were treated operatively, and six had either neurological problems and/or vascular complications; however, none had any long-term neurological compromise, and none required vascular surgical intervention.

Another study published by La O Lafai et al. [19] concluded that 60.8% of fractures (34/56) were treated with reduction and internal fixation with Kirschner wires. It was observed that the treatment received by patients with supracondylar humeral fractures at our hospital is in line with the first six recommendations of AAOS guidelines [9], since type I fractures were treated non-operatively and all displaced fractures were treated with manipulation and stabilization with Kirschner wires.

Regarding associated injuries, Cekanauskas et al. [16] identified preoperative neuropraxia in eight children: four involving the radial nerve, one involving the median nerve, and three involving the cubital nerve. Furthermore, these authors also identified five children with preoperative vascular injuries. Our study showed that associated injuries related to trauma were infrequent. Additionally, severe injuries were very rare, since only two cases required vascular exploration, and there was no case of permanent nerve injury, in line with recommendation number 13 of AAOS guidelines, which states that indication for electromyography is inconclusive, due to lack of evidence of its indication for patients’ follow-up. Since supracondylar humeral fractures occur close to vital structures of the vascular and nervous systems, they have been always related to dreaded complications in these systems. Garland type III fractures were reported to be associated with vascular injuries in 10%-20% of the cases [19-21] and with nerve injuries in approximately 15% [22]. Since 1969, studies have assessed the association between fracture deviations and innervation territories. After anterior interosseous nerve injury was first described by Spinner and Schreiber [23], other nerve branches were associated with directions of deviations: ulnar nerve injuries were found to be related to posterolateral deviations, whereas ulnar, median, and radial injuries are related to posteromedial deviations [22,24,25]. However, all nerve injuries, regardless of innervations territory, typically had revision over time and rarely caused limiting or disabling sequelae that require exploratory or repairing surgeries [26]. In view of the good and excellent results of surgical treatment, currently, there is a consensus that closed reduction and pinning fixation are the most appropriate alternatives to treat displaced fractures [3,6,9].

Rehabilitation with physical therapists or kinesiologists is rarely indicated during patient follow-up (<2%), and recreational physical activities are allowed at six weeks after trauma, once bone union is confirmed by control radiographs obtained during outpatient follow-up.

In Paraguay [27], the first cases of supracondylar humeral fractures in children and adolescents were described by Dr. Hernando Bellasai in 1967. Later, Prof. Dr. Manuel Giagni was the first to apply the Sokolosky tractor for the treatment of supracondylar humeral fractures, with the cooperation of Dr. Víctor I. Franco, Dr. Tulio M. Quirós, Dr. Silvio Allegretti, Dr. Justo R. Podestá, and Dr. Arnaldo Silvero.

We found that it is possible to implement the recommendations suggested by the AAOS at a pediatric trauma referral hospital like ours, since it has the required technology for surgical management, including pediatric intensive care units for cases requiring postoperative management of displaced injuries. Furthermore, its staff includes subspecialists in pediatric orthopedics, as well as a multidisciplinary team (anesthesiologists, vascular surgeons, and plastic surgeons). Therefore, we suggest that the surgical management of pediatric patients with supracondylar humeral fractures should be performed at centers or institutions with the required infrastructure and a team of specialists who can resolve possible complications, and we add a suggestion for doing a future study comparing the treatment of these fractures in different types of hospital centers.

In relation to points 1 and 2 of the AAOS recommendations, we adhere to the suggested approach, as in cases requiring reductions, a vast majority were managed with closed reduction and percutaneous fixation. Concerning the third point of the AAOS guideline, we have very few instances where cross-pinning was performed, following AAOS recommendations as well, which suggest employing cross-pinning when stabilizing the medial column is necessary.

Likewise, in our hospital, we follow the consensus reached in points 7 and 8 of the guideline. In cases of pallor or pulselessness, we carry out manipulation and re-evaluation to determine the definitive course of action.

Regarding the rehabilitation process (points 11 and 12), the AAOS guidelines are inconclusive. Our service chooses not to conduct physical therapy during the process and authorizes recreational activities once fracture consolidation is confirmed through radiographic imaging.

The strength of our study is based on the fact that it is the first study with such a large sample both in our country and in our region. Additionally, it was conducted at a single referral center of a public institution where there is a team of specialists in pediatric orthopedic trauma. Our findings may help in the development of other regional studies or in the comparison of our results with those of other developing countries like ours. Furthermore, our findings make it possible to create national guidelines or recommendations for the management of supracondylar humeral fractures in the pediatric population.

On the other hand, the current study’s limitations stem from its retrospective design, which opens the door to potential biases in the reported outcomes. Moreover, it is crucial to conduct a more comprehensive analysis of factors that could hold significance or act as benchmarks for broader studies. This includes exploring aspects such as the necessity of introducing a third pin to achieve fracture stabilization or considering limitations on the application of medial fixation.

## Conclusions

The present study concluded that supracondylar fractures were more frequent in males and in schoolchildren, affected mostly the left side, occurred more often in the summer, and were mostly caused by falls on the same level. Additionally, the most frequent type of injury was Gartland type III.

The treatment of choice was predominantly closed reduction and percutaneous pin fixation, and a low percentage of immediate and late complications were reported. According to the Mayo Elbow Performance Score, most patients achieved excellent outcomes. The guidelines suggested by the AAOS are complied with at our institution, which is the referral center of a public hospital in a developing country.
